# Early success and safety of three minimally invasive glaucoma surgery techniques combined with cataract surgery in glaucoma patients

**DOI:** 10.1007/s10792-025-03897-5

**Published:** 2025-12-16

**Authors:** Humam Nassri, Julia Prinz, Matthias Fuest, Peter Walter, Niklas Plange, David Kuerten

**Affiliations:** 1 Department of Ophthalmology, University Hospital RWTH Aachen, Pauwelsstraße 30, 52074 Aachen, Germany; 2 Augenzentrum Am Annapark Alsdorf, Steigerweg 3, 52477 Aachen, Germany

**Keywords:** OMNI, VISCO360, Kahook dual blade goniotomy, MIGS, Glaucoma surgery

## Abstract

**Purpose:**

To evaluate the efficacy and safety of three minimally invasive glaucoma surgery (MIGS) techniques, OMNI® Surgical System (OMNI), VISCO360® Surgical System (VISCO) and goniotomy using the Kahook Dual Blade (KDB) in combination with phacoemulsification in glaucoma patients.

**Methods:**

This prospective observational study over 6 months included 93 glaucoma patients who underwent one of the three MIGS combined with cataract surgery. Indications for surgery were insufficient intraocular pressure (IOP) control under topical medication or reducing the medication burden. Complete success was defined as reaching IOP < 21 mmHg without topical medication while qualified success was considered reaching IOP < 21 mmHg with topical medication.

**Results:**

The preoperative IOP was lowered significantly after 6 months for all 3 MIGS procedures and no significant differences in-between the 3 devices were recorded. Topical glaucoma medication was also reduced in all three groups, without a significant difference. Complete surgical success was the highest in KDB patients after 6 months at 74.2%, followed by 58.1% in VISCO and 48.4% in OMNI patients.

**Conclusion:**

OMNI, VISCO or KDB in combination with phacoemulsification are safe and effective treatment options for glaucoma patients. The KDB and VISCO groups achieved higher success rates than OMNI, however, no significant differences in medication burden as well as IOP were recorded in-between the 3 devices.

## Introduction

Glaucoma is one of the leading causes of irreversible blindness in an increasingly aging population [1, 2], especially in developed countries [3]. The prevalence of glaucoma is relatively high; e.g. In Germany the prevalence was found to be 1.4% [4].

Glaucoma causes irreversible damage to the optic nerve head. Lowering the IOP (intraocular pressure) is the only adjustable risk factor today that, as has been proven, slows the progression of glaucomatous damage [5, 6].

IOP results from the interplay between aqueous humor production from the ciliary body, and its drainage through the TM (trabecular meshwork) into the SC (Schlemm’s canal) as well as the alternative uveoscleral outflow pathway [7]. Due to its delicate anatomic structure, the TM is prone to clogging of its fenestrations leading to increased outflow resistance and thereby increase in IOP and subsequent damage to retinal ganglial cells [8]. Furthermore, collapsed SCs have been found in many glaucoma patients and might play a role in increased IOP in some glaucoma patients [9].

In recent years, MIGS (minimally invasive glaucoma surgeries) have become increasingly popular as a safe and effective alternative to traditional filtering surgeries. MIGS aim to improve anatomic drainage into the SC through an ab interno approach, minimizing disturbance of ocular tissue while providing high safety and efficacy, especially in mild to moderate glaucoma cases [10, 11]. Compared to trabeculectomy, MIGS are associated with less side effects and quicker recovery time.

Since the advent of MIGS, cataract surgery is often performed in combination with different MIGS procedures.

The three MIGS in this study include Kahook dual blade excisional goniotomy (KDB), VISCO360® surgical system viscocanaloplasty and OMNI® surgical system viscodilation and trabeculotomy.

KDB entered the market in 2015 as an excisional goniotomy device with promising success rates, especially combined with phacoemulsification (PE) [12]. The most recent version is the second generation KDB GLIDE® with an optimized design that improves performance and operability featuring a sloped edge as well as rounded footplate corners [13].

The VISCO360® surgical system is a technology based on viscodilation of the SC which was developed by Sight Sciences (Inc., Menlo Park, CA, USA). In 2018 Sight Sciences introduced OMNI® which combines viscodilation, using the same instrument in VISCO360®, with trabeculotomy [10].

This study aims to compare the efficacy of the three aforementioned MIGS in combination with cataract surgery in lowering intraocular pressure (IOP), postoperative dependance on IOP-lowering eye drops and safety profile in patients in one surgery center.

## Methods

### Study population

In this prospective observational study 93 patients suffering from primary open angle glaucoma (POAG) or pseudoexfoliative glaucoma (PEX) were included in a 1 to 1 to 1 ratio. All patients underwent one of the following minimally invasive glaucoma surgeries (MIGS): Ab interno canaloplasty and trabeculotomy using OMNI® Surgical System (OSS, Sight Sciences Inc., Menlo Park, CA, USA), ab interno canaloplasty using VISCO360® Surgical System (Viscosurgical System, Sight Sciences, Inc. Menlo Park, CA, USA) or goniotomy using the Kahook Dual Blade (KDB; New World Medical, Rancho, CA, USA) All three procedures were combined with cataract surgery.

The surgeries were performed due to insufficient IOP control under topical medication or to reduce the medication burden on glaucoma patients.

All surgeries were performed at two specialized centers by experienced glaucoma surgeons.

The median age was 72.5 ± 12.8 years for OMNI®, 73.1 ± 8.9 years for VISCO360® and 79.8 ± 5.3 years for KDB. Using Repeated Measures ANOVA with Tukey’s Multiple Comparison Test no significant differences were found in the ages of OMNI vs. Visco patients (*p* < 0.005), whereas the difference in the ages of OMNI®/VISCO360® vs. KDB patients were found to be significant as the latter were older.

All descriptive data is provided in Table 1 for each patient group.

**Table 1:** Study Population

	OMNI®	VISCO360®	KDB	*p* Value
Study eyes	31/93	31/93	31/93	
Age (in years)	72.5 ± 12.8	73.1 ± 8.9	79.8 ± 5.3	** < 0.005**
*Sex*				
• Male • Female	18/31 (58%) 13/31 (42%)	13/31 (42%) 18/31 (58%)	15/31 (48%) 16/31 (52%)	
Medication pre-surgery	2.4 ± 1.4	2.6 ± 1.0	1.7 ± 0.9	** < 0.01**
IOP pre-surgery (in mmHg)	21.9 ± 4.8	20.2 ± 5.3	21.0 ± 3.2	> 0.34
POAG	28/31 (90.3%)	24/31 (77.4%)	25/31 (80.6%)	
PEX	3/31 (9.7%)	7/31 (22.6%)	6/31 (19.4%)	
Prior non-surgical glaucoma interventions	SLT (13/31 = 41.9%) ALT (2/31 = 6.5%)	SLT (2/31 = 6.5%)	SLT (6/31 = 19.4%)	
Axial Length (in mm)	23.6 ± 0.9	23.9 ± 1.4	23.2 ± 0.5	> 0.06
Cup to disc ratio	0.72 ± 0.19	0.76 ± 0.14	0.72 ± 0.2	> 0.41

POAG = Primary open angle glaucoma; PEX = Pseudoexfoliative glaucoma; SLT = Selective Laser Trabeculoplasty; ALT = Argon Laser Trabeculoplasty. Mean and standard deviation is shown

Statistically significant differences are highlighted in bold print

### Inclusion and exclusion criteria

Inclusion criteria for this study required subjects to be of full age and have had a previously confirmed diagnosis of glaucoma and/or significant glaucomatous damage based on optic nerve head evaluation, intraocular pressure (IOP) measurement and automated static perimetry. Different types of glaucoma were included such as primary open angle glaucoma (POAG) as well as PEX (pseudoexfoliative glaucoma).

Exclusion criteria included prior glaucoma surgery as well as aphakic patients.

All eyes underwent one of the three MIGS mentioned above.

Subjects who missed their 2- and 6-months follow up appointments were excluded from the study, luckily no participant in this study had to be excluded due to missing data.

### Surgical techniques

Prior to each MIGS, a cataract surgery was performed using phacoemulsification and the implantation of a foldable acrylic intraocular lens (ASPIRA-aA, Humanoptics, Erlangen, Germany) into the lens capsule.

After completion of cataract surgery acetylcholine chloride 1% (Miochol-E, Dr. Gerhard Mann, Berlin, Germany) was instilled intracamerally to contract the pupil. Next, a cohesive ophthalmic viscoelastic (ALBOMED GmbH, Germany) was used to deepen the anterior chamber (AC). The AC angle was visualized in all three procedures with the help of a direct gonioscope (MV LV 48, Phakos, Montreuil, France). Thereafter, one of the following surgical systems was used to perform the MIGS, which all have the ab interno approach in common.

#### Kahook dual blade excisional goniotomy

The Kahook dual blade (KDB; New World Medical, Rancho, CA, USA) was used to resect strips of the trabecular meshwork (TM) and thereby boost the outflow of aqueous humor and consequently lower the IOP. The KDB was inserted through the temporal corneal incision made for cataract surgery. Afterwards the blade’s cut through the trabecular meshwork (TM) into the Schlemm’s canal (SC) began midnasal and was conducted firstly in the inferior hemisphere. The baseplate of KDB was positioned against the inner wall of the SC, the sharp head was pushed along the TM in an anticlockwise direction for 90 degrees. Due to the dual parallel blades of the KBD, paired parallel incisions were produced and thereby a strip of TM was excised. Afterwards the KDB was turned, and the same procedure was executed in the superior hemisphere. In case of an immediate hyphemia, the AC was irrigated, so that no postoperative intervention was necessary.

#### VISCO360® surgical system viscocanaloplasty

The VISCO360® (Viscosurgical System, Sight Sciences, Inc. Menlo Park, CA, USA) surgical system uses an ab interno approach as well through the clear corneal incision which has been initially made for cataract surgery. With the cannula, the tip of the VISCO360® microcatheter, a small incision of the TM was made to reach the SC at the iridocorneal angle. The catheter was then advanced 180 degrees through the TM. Furthermore, a controlled volume of viscoelasticum (Healon GV, AMO Germany GmbH) is injected into the SC which dilates the canal. Then this process was repeated on the other 180 degrees to achieve a complete 360-degree viscodilation of the SC.

#### OMNI® surgical system viscodilation and trabeculotomy

OMNI® surgical system (OSS, Sight Sciences Inc., Menlo Park, CA, USA) utilized a two-step ab interno approach by combining viscodilation and subsequently a 360-degree trabeculotomy to restore the function of the SC and facilitate the outflow of aqueous humor.

After the cataract surgery, the OSS microcatheter was advanced into the SC, which was dilated with viscoelasticum. At the end of the viscodilation with Healon GV (AMO Germany GmbH) the microcatheter was threaded through the canal to perform a 360-degree trabeculotomy in two 180° steps (comparable to the VISCO 360 surgery) to further enhance outflow by incising the trabecular meshwork. The OMNI is the recent advancement of the VISCO 360 system and has substituted the previously used VISCO 360 system.

At the end of each surgery the corneal incisions were hydrated and checked for water-tight closure. Postoperatively dexamethasone and gentamicin eyedrops were applied five times daily for the first week and reduced subsequently in the following week.

### Patient allocation

Patient allocation to the different surgical techniques was not randomized and done by the individual surgeons digression. The patients were asked to participate in the study in a 1:1:1 ratio.

### Outcome measure

The data gathered one week to one day prior to the surgery as well as 2- and 6- months post-surgery, encompasses IOP, IOP-lowering medication (number and type of medication), automatic static perimetry using the mean deviation in decibels (Zeiss Octopus 900, Zeiss, Germany) (only one day prior and 6 months after surgery), visual acuity as well as complications intra- and post-operatively. Demographics (age at surgery, sex, affected eye) were also included as well as preoperative cup to disc ratio (CDR). IOP measurements were taken between 9 and 12am by goldmann applanation tonometry (GAT) in all patients.

Success of the respective surgical interventions was divided into complete and qualified success. Complete success was defined as achieving target IOP of 21 without the need for IOP-lowering medication post-surgery. Qualified success was considered reaching those same IOP values with the use of antiglaucoma medication. The IOP values were measured using Goldmann applanation tonometry prior to surgery as well as 2 and 6 months after surgery.

Furthermore, the number and type of topical IOP-lowering medication were assessed. Adverse effects were documented post-operatively after 2 and 6 months as well as immediately during the first two postoperative weeks. The occurrence of a fibrin reaction, hyphemia, vitreous hemorrhage, Irvine-Gass syndrome, macular edema and IOP crisis were especially evaluated and considered adverse events.

The encountered complications were recorded and are presented in Table 2.

**Table 2:** Surgical Complications

OMNI®	VISCO360®	KDB
• Hyphema (1/31 = 3.2%) • Transient IOP rise (2/31 = 6.4%) • Transient Hypotonie (1/31 = 3.2%) • Fibrin reaction (1/31 = 3.2%) • Vitreous hemorrhage (1/31 = 3.2%)	• Hyphema (2/31 = 6.4%) • Fibrin reaction (3/31 = 9.6%) • Transient IOP rise (2/31 = 6.4%)	• Fibrin reaction (2/31 = 6.4%)

### Statistical analysis

The statistical analysis as well as graph creation was conducted using Graphpad Prism, SPSS as well as Excel. For further analysis repeated measures ANOVA with Tukey’s multiple comparison test was performed, if applicable. An ANCOVA was performed to determine the influence of Age, prior medication and preoperative IOP in the different groups.

## Results

Regarding functional data, following values were recorded.

The visual field MD in dB was -5.6 ± 6.7, -6.2 ± 2.9, -5.0 ± 4.1 for OMNI, VISCO360 and KDB respectively and changed to -5.2 ± 6.3, -6.0 ± 4.43, -5.7 ± 3.8 after 6 months. None of the changes was statistically significant (paired t-test. *p* > 0.23, > 0.48 and *p* > 0.13 respectively).

Decimal visual acuity improved significantly from 0.66 ± 0.22, 0.45 ± 0.24 and 0.55 ± 0.25 prior to the surgery for OMNI, VISCO360 and KDB respectively, to 0.86 ± 0.16, 0.67 ± 0.25, 0.87 ± 0.12. 2 months after surgery (*p* < 0.0008 for all 3 interventions) and did not change for the following observation period.

**Fig. 1:**
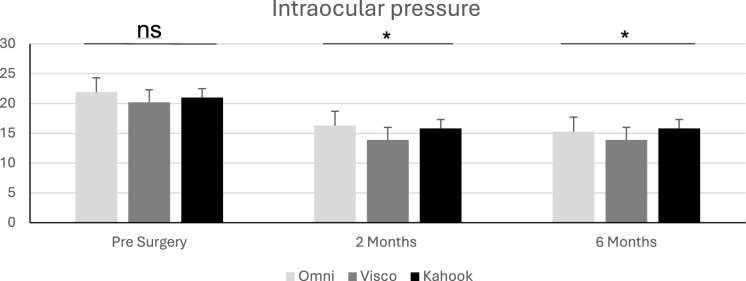
Demonstrating the intraocular pressure over the course of the observation for OMNI®, VISCO360® and KDB patients (ns = not significant; * = significant)

No significant differences were recorded prior to the surgery and IOP was significantly reduced in all 3 groups. At two months a significant difference was recorded in all 3 groups. OMNI® (16.3 ± 4.8 mmHg) and VISCO360® (13.9 ± 3.1 mmHg) differed significantly from another, whereas both techniques did not differ significantly from the KDB (15.8 ± 2.3 mmHg) group (post-hoc analysis). At 6 months no significant differences between the 3 groups were recorded (*p* > 0.09). The OMNI® group had an IOP of 15.3 ± 4.8 mmHg, the VISCO360® group 13.9 ± 2.7 mmHg and the KDB group 15.8 ± 2.6mmHg. Please refer to Fig. 1 for visualization. The ANCOVA analysis revealed that only preoperative IOP was significantly affecting IOP at 6 months (F = 5.3, *p* < 0.03), neither Age (F = 1.5, *p* > 0.2) nor preoperative medication (F = 3.9, *p* > 0.05) affected IOP at 6 months significantly.

In terms of glaucoma medication a significant difference was found between the 3 groups prior to the surgeries. The KDB patients were taking significantly less medications (1.7 ± 0.9) than either the VISCO360® (2.6 ± 1.0) or OMNI® (2.4 ± 1.4) patients (*p* < 0.01 and post-hoc analysis). Two months after surgery another significant difference was recorded in terms of glaucoma medication. The OMNI® patients were taking significantly more medication (1.4 ± 1.1) than either the VISCO360® (0.7 ± 1.2) or KDB patients (0.5 ± 0.8) respectively, the KDB and VISCO360® group did not differ significantly (p < 0.001 and post hoc analysis). 6 months after surgery no significant differences regarding antiglaucoma medication were recorded. The OMNI® patients took (1.0 ± 1.3), the VISCO360® patients (0.9 ± 1.3) and the KDB patients (0.4 ± 0.8) (*p* > 0.06). The ANCOVA analysis showed that only prior medication significantly correlated to postoperative medication (F = 14.8, *p* < 0.005), neither age (F = 0.02, *p* > 0.8) nor preoperative IOP (F = 0.6, *p* > 0.4) affected medication at 6 months.

No significant complications were encountered in our study. The complications were transient and self-limiting. All complications are presented in Table 2.

1 patient in the VISCO360® group was deemed a surgical failure with the need for another intervention prior to completion of the 6-month observation period and a XEN-Stent was implanted two months after failing the initial surgery.

Other parameters are presented in Table 3.

**Table 3:** IOP and medication reduction, surgical success

	OMNI®	VISCO360®	KDB
IOP reduction to baseline 2 months after surgery (in %)	23.3 ± 24.5	31.1 ± 21.8	24.1 ± 11.0
IOP reduction to baseline 6 months after surgery (in %)	30.1 ± 25.6	30.1 ± 24.6	23.6 ± 14.4
Medication reduction to baseline 2 months after surgery	1.0 ± 1.3	1.9 ± 1.3	1.29 ± 1.1
Medication reduction to baseline 6 months after surgery	1.4 ± 1.3 (65.5 ± 41.85%)	1.7 ± 1.4 (71.9 ± 44.9%)	1.4 ± 1.1 (78.1 ± 40%)
Complete surgical success 2 months after surgery (in %)	25.8	67.7	67.7
Complete surgical success 6 months after surgery (in %)	48.4	58.1	74.2
Qualified surgical success 2 months after surgery (in %)	83.8	93.5	100
Qualified surgical success 6 months after surgery (in %)	87.1	96.8	100

Complete surgical success was defined as an IOP < 21 mmHg without the need for topical medication. Qualified success was defined as an IOP < 21 mmHg with topical medication

In OMNI®-treated eyes, a reduction in IOP was observed postoperatively, from a mean preoperative IOP of 21.9 ± 4.8 mmHg, taking 2.4 ± 1.4 antiglaucoma medications, to a mean IOP of 16.3 ± 4.8 mmHg, taking 1.4 ± 1.1 medications, 2 months postoperatively. At 6 months the IOP further declined to 15.3 ± 4.8 mmHg. Additionally, there was a noteworthy reduction in the number of anti-glaucoma medications from the average of 2.4 ± 1.4 pre-operatively to 1.4 ± 1.1 at two months and to 1.0 ± 1.3 at six months (see Table 2 for further details).

**Fig. 2:**
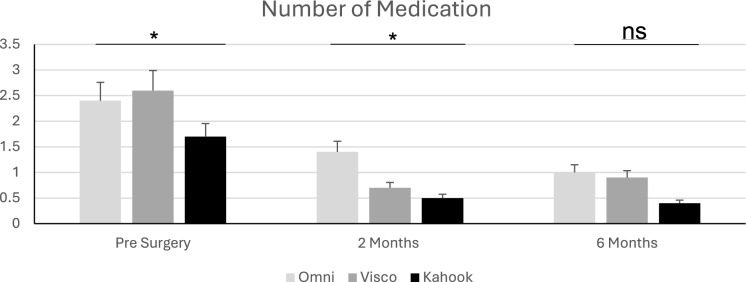
Demonstrating the number of medications taken over the observation period in OMNI®, VISCO360® and KDB patients (ns = not significant; * = significant)

In VISCO360® eyes the IOP decreased 2 months post-operatively from a mean preoperative IOP of 20.2 ± 5.3 mmHg to 13.9 ± 3.1 mmHg at months. 6 months after the procedure the mean IOP remained at 13.9 ± 2.7 mmHg, not declining further. In parallel, the number of medications required for IOP management initially decreased from 2.6 ± 1.0 preoperatively to 0.7 ± 1.2 at 2 months, which rose slightly to 0.9 ± 1.3 at 6 months. Please refer to Fig. 2 for visualization.

KDB surgery resulted in a drop from a preoperative IOP of 21 ± 3.2 mmHg to 15.8 ± 2.3 mmHg. At the 6 months observation visit the mean IOP value remained at 15.8 mmHg with a standard deviation of 2.6. The number of IOP-lowering drugs was lessened from a preoperative value of 1.7 ± 0.9, compared to VISCO360® and OMNI® patients, to 0.5 ± 0.8 at 2 months and to 0.4 ± 0.8 at 6 months post-operatively. The relative reduction in medications was 78.1% ± 40% at 6 months.

The medication used 2 months post-operatively comparing OMNI® vs VISCO360® eyes (1.4 ± 1.1 and 0.7 ± 1.2) as well as OMNI® vs KDB (1.4 ± 1.1 and 0.5 ± 0.8) were found to be significantly different (*p* < 0.001). However, comparing mean values of medication used at 2 months in VISCO360® vs KDB no significant difference was found, at 0.7 and 0.5, respectively. In all three groups the IOP values at 6 months were not found to be significantly different, as was the case regarding the mean number of medications at 6 months in all three groups (*p* > 0.09, *p* > 0.06, respectively; Repeated Measures ANOVA with Tukey’s Multiple Comparison Test).

## Discussion

This is the first study comparing the efficacy and safety of the three MIGS procedures OMNI®, VISCO360® and KDB, combined with PE, in similar patient populations and performed by the same surgeons from two academic ophthalmology centers. All three procedures can effectively and safely lower IOP as well as reduce the medication burden in patients with mild to moderate glaucoma, observed over the period of 6 months post-operatively. Moreover, complete success after 6 months, defined as reaching an IOP < 21 mmHg without the need for additional IOP lowering medication, was achieved in by 48.4% of OMNI®, 58.1.% of VISCO360® and 74.2% of KDB patients.

While in our study the OMNI® group achieved a 30.1% IOP decrease from baseline at 6 months, a study conducted by Toneatto et. al in 2022 showed an IOP decrease by 30.3% 6 months post-operatively in the OMNI + PE group [14]. And a prospective study published in 2021 demonstrated an IOP decrease by 25% after 6 months in the group undergoing OMNI and PE [15].

VISCO360®-treated patients experienced a 30.1% decline in IOP at 6 months post-operatively which is lower than a retrospective analysis of 106 conducted by Ondrejka et al. in 2019 presenting a 44.3% IOP reduction (from 24.6 to 13.7 mmHg) 6 months after VISCO360 surgery with or without PE in group 1 patients which included 72 eyes with a baseline IOP ≥ 18 mmHg [16]. In another retrospective consecutive case series, the IOP was lowered from 24.5 to 17.2 mmHg (equivalent to a 29.7% decrease) 6 months after a standalone Visco360® procedure or combined with PE, which is comparable with our findings [17].

Furthermore, KDB patients achieved a 23.6% reduction in IOP at 6 months similar to previously published data by Baumgarten et. with an IOP reduction of 21% at 6 months post-operatively [18]. Besides, we were able to publish a 15.3% IOP lowering at 6 months post-surgically [11].

All three procedures lowered the IOP and medication burden significantly in the first 6 months, and the decrease was comparable to multiple previous studies concerning all three surgical techniques. Interestingly, in our study VISCO360® proved to be the most effective surgical technique in the early postoperative stage with a 31.1% IOP reduction as well as 67.7% of patients achieving complete surgical success (reaching < 21 mmHg without medication) at 2 months post-operatively. This might indicate that the collapse of SC is partly responsible for the high IOP.

The medication burden was reduced the most in VISCO360® patients compared to OMNI® and KDB, initially by 1.9 ± 1.3 after 2 months, then by 1.7 ± 1.4 at 6 months post-surgically. However, no significant differences were found in the number of medications after 6 months among the three groups. This finding is probably biased, since the number of preoperative topical medications in the KDB group was lower than in the other two groups and thereby the reduction in absolute medication was lower. Nevertheless the relative reduction in medication burden, which was the highest in the KDB group with 78% at 6 months compared to 65.5% in OMNI and 71.9% in the VISCO group, reaffirming the high success rates found in the KDB group. ANCOVA analysis revealed that only preoperative influenced the number of medications at 6 months.

Several studies showcased the safety and short-term as well as long-term effectiveness of OMNI®, such as a retrospective study conducted by Bleeker et. al in 2022 and the ROMEO study published in the Clinical Ophthalmology Journal (V. 16 and 17), respectively. Bleeker et. al demonstrated 29% mean decrease in IOP 6 months after the surgery while 69.8% of patients achieved surgical success which was defined as ≥ 20% IOP reduction from baseline without an increase in the number of glaucoma medications or decrease of at least one medication with no increase in IOP [19].

Similarly, the ROMEO study showed 28.2% mean IOP reduction 6 months post-operatively in group 1 patients (defined as IOP > 18 mmHg, OMNI with PE) along with 81.8% of group 1 achieving surgical success after 6 months (termed primary effectiveness in their study) which was defined as reaching a 20% fall in IOP from baseline or an IOP ≤ 18 mmHg [20].

Even though OMNI® achieved the lowest complete success rate in our study, the number of patients who underwent OMNI® with complete surgical success nearly doubled from 25.8% at 2 months to almost fifty percent at 6 months.

In contrast to the increase in success in the OMNI patients, the percentage of VISCO360® patients with complete success declined from 67.7% at 2 months to 58.1% at 6 months. Therefore, the additional benefits of incising the trabecular meshwork seem to be providing a benefit over time and show worse results in the early stages. We are interested to see if this assumption is proven by longer-term investigations in a head to head comparison.

In the KDB group, complete success was achieved initially by 67.7% and slightly rose to 74.2% and was the highest in our study. Nevertheless, the number of medications taken by the KDB patients was significantly lower in our study, thereby it has probably been easier to achieve complete surgical success by the defined criteria. Nevertheless, the KDB and PE procedure also provided the highest relative medication reduction in our study as well, highlighting its effectiveness. A retrospective study published in 2018 at the American Glaucoma Society Annual Meeting presented a success rate (defined as ≥ 20 IOP decrease from baseline or reduction of at least 1 glaucoma drug) of 71.8% at 12 months post-operatively for 165 eyes that underwent KDB with cataract surgery [21].

Regarding the qualified success, defined as reaching an IOP of < 21 mmHg with the use of antiglaucoma drugs, the VISCO360® and KDB group reached 97–100%, whereas the OMNI® group reached only 87%.

In conclusion, all three MIGS can be conducted to reduce the IOP effectively in glaucoma patients and decrease the number of topical antiglaucoma medication. The KDB and VISCO360® group achieved higher success rates than the OMNI® group after six months. It was rather surprising for us, that in the early postoperative period OMNI did not perform better than its’ predecessor VISCO360. The additional effect of incising the trabecular meshwork does not seem to provide a benefit in the first 6 months after surgery in our patients. Overall more complications were recorded in these patients, which seems natural, as the incision of trabecular meshwork for almost 360° is an additional surgical step. We are interested to see, if the same trend persists in a longer observation period. Interestingly, KDB was able to achieve positive results as well. It did not perform worse than the other angle chamber techniques, which have a 360°-degree angle of action, whereas with the KDB only a limited area of trabecular meshwork is incised (about 90–120°). Maybe the excision of trabecular meshwork by the KDB is able to reestablish flow into the SC and consecutively widens it. However further studies are necessary, if any effect on the SC can be detected after successfully KDB. A study by Strohmaier et al. highlighted, that targeted trabecular bypass surgery in low flow regions did increase IOP reduction [22].It might be, that these areas are often found in the nasal region coinciding with the incision side in KDB surgery and thereby explaining the advantageous results in many studies.

Our study was never specifically aimed at detecting differences between the 3 devices, and the power of our study appears to be insufficient to do so, therefore all comparisons between the devices should not be generalized.

### Limitations

When interpreting the results of this study and particularly the interpretation of the results with previous data, it is of utmost importance to discuss the issue with all MIGS procedures, which is the limited data available to date. It is still not clear to data if data of a MIGS procedure as a standalone procedure is really comparable to a combination of a MIGS procedure with PE. It was previously shown that PE can lower IOP effectively in glaucoma patients [23].Thereby comparisons of our findings with previously published results, where the interventions were used as a standalone procedure, might not be completely accurate and should only be considered a ballpark figure.

Furthermore, it is crucial to consider the limitations encountered which include a relatively small number of patients from all 3 groups. As seen in our study, whereas the IOP prior to the surgery did not differ significantly in-between the groups, medication and age did. Although ANCOVA analysis revealed to influence of age on either medication or IOP at 6 months, IOP and medication influenced their respective outcome parameter significantly. Larger, more homogenous groups with randomized allocation are desirable in the future.

In addition, the observation period of only 6 months is rather short, particularly in an often slowly progressing disease like glaucoma and longer-term studies are warranted and currently in progress. More long-term Omni studies with a larger patient population are necessary as the current long-term data is sparse to date, in order to observe the efficacy of OMNI® compared to other MIGS. The Gemini study, for instance, conducted in 2023 demonstrated that 77% of their patients undergoing OMNI® surgery have achieved ≥ 20% IOP lowering after 24 months and 78% after 36 months of post-surgical observation. Additionally, 74% were medication-free after 36 months, showcasing the sustained efficacy of OMNI® over a prolonged period, however the number of patients included in the Gemini study is also rather sparse [24]. Further, the typical disadvantages of non-randomized studies apply on our study as well, particularly the possibility of a selection bias. The VISCO surgeries were performed by the same experienced surgeon, whereas the OMNI and Kahook surgeries were performed by all surgeons. KDB was often performed in elderly patients due to its fast surgery time (fastest of the 3 surgical techniques in this study), perceived less invasive nature (which is somewhat reflected in the lowest number of complications encountered in our study, please refer to Table 2) and being the easiest to perform. Furthermore, the examiners for the post-operative controls were not masked to the used intervention, nevertheless the examinations were not performed by the surgeons themselves, which might have added another bias.

Moreover, the surgeries were performed by specialized surgeons at two centers thus the results may differ in other surgical centers. The decision for combined surgery was made to lessen the burden for the patients and create a similar setting to most combined surgeries performed in the US. The results in our study are dependent on our surgeons and surgical set-ups, therefore they might not be transferable to other surgical centers all over the globe.

Furthermore the number of PEX-patients differed in the 3 groups, whereas these patients often tend to show lower success rates after glaucoma surgery. In the short-term investigation this did not seem to be the case, however long-term data is necessary. Correlation analysis showed no effect of PEX on the outcome in our study, however the small sample size hinders a meaningful analysis.

## Data Availability

The anonymized data scales used to support the findings of this study are available from the corresponding author upon reasonable request.
